# Norepinephrine Transporter (SLC6A2) Polymorphisms and their Impact on SSRI/SNRI Treatment response in Indian Patients with Depressive Disorder: A preliminary study

**DOI:** 10.1192/j.eurpsy.2026.10584

**Published:** 2026-07-31

**Authors:** S. Sinha, S. C. Sarangi, D. Sharma, B. N. Patra, S. Singh, R. Sagar

**Affiliations:** 1 Pharmacology; 2 Psychiatry, All India Institute of Medical Sciences, New Delhi, India

## Abstract

**Introduction:**

Treatment-resistant depression is a major concern, though there has been significant advancement in antidepressant therapy. The therapeutic outcome for patients with depressive disorder is achieving a treatment response with remission. Predictions based on genetic markers may have the potential to increase the treatment response through a personalised approach. Norepinephrine neurotransmission is a major pathophysiological concern.

**Objectives:**

To investigate the association of single-nucleotide polymorphisms (SNPs) in the norepinephrine transporter (SLC6A2) as predictive pharmacogenetic biomarkers with antidepressant response in the Indian population, by comparing treatment responders and non-responders to SSRI/SNRI monotherapy.

**Methods:**

Two hundred thirty-eight patients suffering from depressive disorder were selected and enrolled for this study. The patients were treated with SSRI/SNRI over a period of 3 months. Standardised questionnaires were used for assessment, including the severity of depression assessed by the Hamilton Depression Rating Scale (HDRS) and the Centre for Epidemiologic Studies Depression Scale (CES-D), social cognition assessment using SOCRATIS, and side effects evaluation with the UKU Side Effect Rating Scale. Genetic variations SLC6A2 with their SNPs (rs5569 and rs36029) were genotyped. The genetic data of the responders and non-responders were compared to evaluate the role of genetic variants in therapeutic outcome.

**Results:**

Out of the total patients, 136 (57.14%) showed a positive treatment response, with 32 (23.52%) achieving complete remission. Analysis of SNP rs36029 (A/G) revealed no significant association with responder/non-responder or remitter/non-remitter groups, nor with the eight most common side effects, except memory loss, which showed a significant (p = 0.029). For SNP rs5529 (G/A/C), the wild-type allele (G) was predominant among responders (56 patients, 37.3%), while mutant alleles A (19 patients, 12%) and C (1 patient, 0.7%) were less common. In non-responder groups, the G allele was less frequent (34 patients, 12.5%), with higher prevalence of A (29 patients, 17.3%) and C (11 patients, 7.3%). These findings suggest that mutant alleles, especially C, may reduce treatment efficacy. **Notably, the NET rs5569 allele C has not been previously reported in the Indian population.**

**Image 1:**
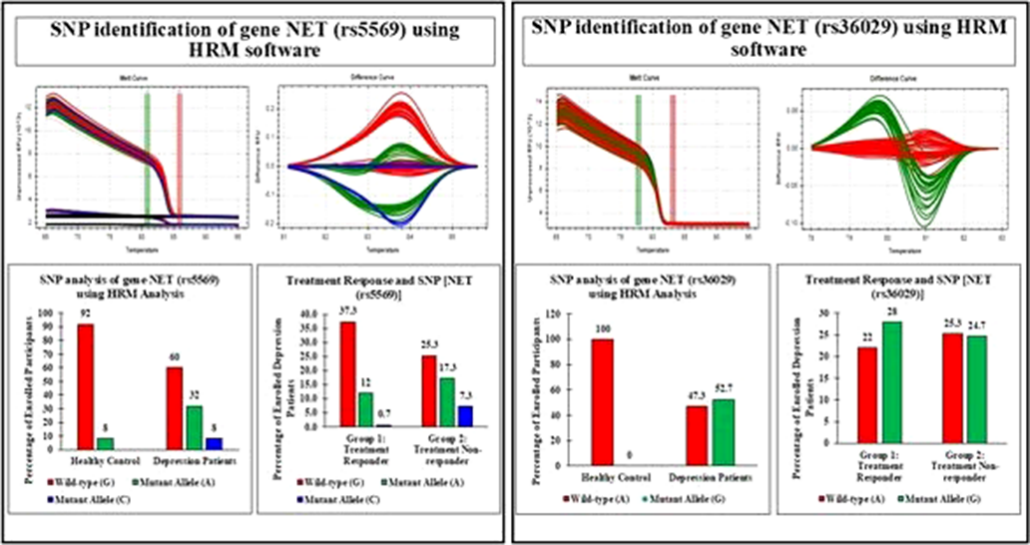
Image 1: Long description.

**Conclusions:**

This study suggests that while SNP rs36029 (A/G) showed no association with treatment response, it was significantly linked to memory loss. In contrast, SNP rs5529 (G/A/C) indicated that mutant alleles, particularly allele C—reported for the first time in the Indian population—may contribute to reduced antidepressant efficacy. Studies with larger samples are required to find out the genetic basis of antidepressant response in Indian patients.

**Disclosure of Interest:**

None Declared

## Long descriptions

### Image 1: Long description

The figure is divided into two large panels.

Left Panel: S N P identification of gene N E T (rs 5 5 6 9).

- Top row: Two line graphs. The first is a Melt Curve showing fluorescence decreasing as temperature increases from 65 to 90 degrees Celsius. The second is a Difference Curve showing three distinct clusters of curves (red, green, and blue) representing different genotypes.

- Bottom row: Two bar charts. The left chart shows S N P analysis for Healthy Controls (92 percent Wild-type G, 8 percent Mutant Allele A) and Depression Patients (60 percent Wild-type G, 32 percent Mutant Allele A, 8 percent Mutant Allele C). The right chart shows Treatment Response, where Group 1 (Responders) has 87.3 percent Wild-type G and Group 2 (Non-responders) has 75.3 percent Wild-type G.

Right Panel: S N P identification of gene N E T (rs 3 6 0 2 9).

- Top row: Similar Melt and Difference curves. The Difference Curve shows two primary clusters (red and green).

- Bottom row: Two bar charts. The left chart shows Healthy Controls are 100 percent Wild-type A, while Depression Patients are 47.3 percent Wild-type A and 52.7 percent Mutant Allele G. The right chart shows Treatment Response for Group 1 (22 percent Wild-type A, 28 percent Mutant Allele G) and Group 2 (25.3 percent Wild-type A, 24.7 percent Mutant Allele G).

Navigate back to Image 1:.

